# Biomarkers for a histological chorioamnionitis diagnosis in pregnant women with or without group B streptococcus infection: a case-control study

**DOI:** 10.1186/s12884-021-03731-7

**Published:** 2021-03-25

**Authors:** Jie Ren, Zhe Qiang, Yuan-yuan Li, Jun-na Zhang

**Affiliations:** 1 Second Department of Obstetrics, The Fourth Hospital of Shijiazhuang, No.206, Zhongshan East Road, Chang’an District, Shijiazhuang, Hebei People’s Republic of China 050011; 2 Perinatal center, The Fourth Hospital of Shijiazhuang, Shijiazhuang, Hebei People’s Republic of China 050000

**Keywords:** Chorioamnionitis, Group B streptococcus, Biomarkers, Diagnosis, Case-control

## Abstract

**Background:**

Chorioamnionitis may cause serious perinatal and neonatal adverse outcomes, and group B streptococcus (GBS) is one of the most common bacteria isolated from human chorioamnionitis. The present study analyzed the impact of GBS infection and histological chorioamnionitis (HCA) on pregnancy outcomes and the diagnostic value of various biomarkers.

**Methods:**

Pregnant women were grouped according to GBS infection and HCA detection. Perinatal and neonatal adverse outcomes were recorded with a follow-up period of 6 weeks. The white blood cell count (WBC), neutrophil ratio, and C-reactive protein (CRP) level from peripheral blood and soluble intercellular adhesion molecule-1 (sICAM-1), interleukin 8 (IL-8), and tumor necrosis factor α (TNF-α) levels from cord blood were assessed.

**Results:**

A total of 371 pregnant women were included. Pregnant women with GBS infection or HCA had a higher risk of pathological jaundice and premature rupture of membranes and higher levels of sICAM-1, IL-8, and TNF-α in umbilical cord blood. Univariate and multivariate regression analysis revealed that sICMA-1, IL-8, TNF-α, WBC, and CRP were significantly related to an increased HCA risk. For all included pregnant women, TNF-α had the largest receiver operating characteristic (ROC) area (area: 0.841; 95% CI: 0.778–0.904) of the biomarkers analyzed. TNF-α still had the largest area under the ROC curve (area: 0.898; 95% CI: 0.814–0.982) for non-GBS-infected pregnant women, who also exhibited a higher neutrophil ratio (area: 0.815; 95% CI: 0.645–0.985) and WBC (area: 0.849; 95% CI: 0.72–0.978), but all biomarkers had lower value in the diagnosis of HCA in GBS-infected pregnant women.

**Conclusion:**

GBS infection and HCA correlated with several perinatal and neonatal adverse outcomes. TNF-α in cord blood and WBCs in peripheral blood had diagnostic value for HCA in non-GBS-infected pregnant women but not GBS-infected pregnant women.

**Supplementary Information:**

The online version contains supplementary material available at 10.1186/s12884-021-03731-7.

## Background

Chorioamnionitis refers to neutrophil-related inflammation in the placental and umbilical cord tissue due to microbial infection, which may cause adverse pregnancy outcomes, including premature birth, premature rupture of membranes, puerperal infection, and sepsis. The recommended term “chorioamnionitis” should be used instead of “intrauterine infection” [1]. Typical clinical symptoms include maternal body temperature > =38 °C, purulent fluid from the vagina with a peculiar smell, a baseline fetal heart rate > =160 times per minute, a maternal peripheral white blood cell count (WBC) ≥15 × 10^9^, uterine irritation, and a tender uterine body [2]. However, clinical symptoms often occur in late-stage pregnancy, and typical clinical chorioamnionitis have become less common with improvements in perinatal medical conditions. Occult chorioamnionitis, also known as histological chorioamnionitis (HCA), is diagnosed during the pathological examination of the placenta. These factors may cause pregnant women to not receive a timely diagnosis and treatment, which may affect the health of the mother and baby. Therefore, early diagnosis is essential.

Infection is the main cause of chorioamnionitis, and group B streptococcus (GBS) is one of the most common bacteria isolated from human chorioamnionitis [3, 4]. The mean prevalence of rectovaginal GBS colonization is 17.9% (95% confidence interval (CI): 16.2–22%), of which 22.4% (95%CI: 18.1–26.7%) occurs in Africa, followed by 19.7% (95%CI:16.7–22.7%) in the Americas and 19% (95%CI: 16.1–22.0%) in Europe [5]. GBS is a β hemolytic, Gram-positive bacterium that generally colonizes the rectum and vagina of a healthy woman, but when infection occurs during childbirth, there is an approximately 36% probability of transmission to the newborn [6]. GBS may exhibit mass propagation and transmission in utero via contaminated vaginal or amniotic fluid pumping [7, 8], which leads to platelet consumption, pathological coagulation (such as disseminated intravascular coagulation) and severe bacterial sepsis [9]. Neonatal invasive GBS infection is divided into two forms: early-onset disease (EOD) and late-onset disease (LOD). EOD generally occurs in the first week after birth, with pneumonia and respiratory failure accompanied by blood infections, sepsis and low-probability meningitis. LOD occurs within 7 months with bacteremia and higher incidence of meningitis [10]. However, most newborns with GBS infection are asymptomatic, and only approximately 3% of newborns have EOD [6]. Although antibiotics are routinely used to prevent infection and are effective for EOD, well-designed randomized controlled trials (RCTs) showed that antibiotics application did not significantly reduce the incidence of HCA [11–13].

The present analyzed the impact of GBS infection on pregnancy outcomes and biomarker levels and evaluated the influence of HCA on pregnancy outcomes and the diagnostic value of various biomarkers.

## Methods

This case-control research followed the strengthening of the reporting of observational studies in epidemiology reporting guidelines. This study enrolled pregnant women who had undergone prenatal checkups and delivery in local hospitals from May 2019 to March 2020. A survey was conducted in this study (Supplementary file).

### Inclusion and exclusion criteria

The following inclusion criteria were used: 1, gestational age greater than 36 weeks; 2, no antibiotic use in the previous 4 weeks; 3, routine GBS infection detection before delivery; 4, singleton pregnancy; and 5, availability of cord blood, umbilical cord, and placenta after delivery. The following exclusion criteria were used: 1, people who under 18 years old; 2, people who lacked any GBS infection test before childbirth, cord blood, umbilical cord, or placenta collection after childbirth; 3, people who had serious non-GBS infection diseases; 4, people with malignant tumor; 5, hypertension or diabetes population; 6, genital malformation; 7, people with nephritis; and 8, habitual abortion. The study did not limit the gravidity and parity history.

### GBS infection detection

Women were divided into GBS infection and non-GBS infection groups based on the occurrence of GBS infection. Vaginal and perianal secretion samples were obtained from all women at 36–38 weeks of gestation for GBS culture. After removal of excess secretions, a sterile cotton swab was used to collect samples from the lower 1/3 of the vagina and 3 cm inside the anus. Patients who were GBS-culture positive were classified into the GBS infection group and treated with antibiotics for preventive treatment. Penicillin G was the first choice of treatment, at an initial dose was 5 million units, followed by 2.5 million units every 4 hours until delivery. Cefazolin was used in cases of penicillin allergy. The initial dose was 2 g, followed by 1 g every 8 hours until delivery. Clindamycin was used at 900 mg every 8 hours until delivery in cases of penicillin and cefazolin allergy and bacterial sensitivity to clindamycin. If the bacteria was resistant to clindamycin, then vancomycin was used at 20 mg/kg every 8 hours until delivery.

### Clinical definitions

Pathological jaundice was defined as any of the following features: clinical jaundice appearing within the first 24 h or after 14 days of life; increases in the levels of total bilirubin by > 8.5 μmol/L (0.5 mg/dL) per hour or (85 μmol/L) 5 mg/dL per 24 h; total bilirubin > 331.5 μmol/L (19.5 mg/dL); direct bilirubin > 34 μmol/L (2.0 mg/dL), which primarily referred to the National Institute for Health and Care Excellence (NICE) guideline and Queensland Clinical Guidelines. Non-reassuring fetal status was defined as progressive fetal hypoxia and/or acidemia secondary to inadequate fetal oxygenation, and it was diagnosed when any one of the following conditions was met: II-III-degree amniotic fluid meconium-staining; a fetal heart rate > 160 beats per minute or < 120 beats per minute for > 10 min; an abnormal fetal heart rate (repetitive late decelerations, undulating baseline, bradycardia); or fetal movement <three times per hour or an increase or decrease of 50% compared to the previous frequency of fetal movements, with no recovery after repeated observations, which primarily referred to the National Institute of Child Health and Human Development (NICHD) guidelines [14, 15]. Birth asphyxia was defined as failing to initiate and sustain breathing at birth. Moderate birth asphyxia was diagnostic when at least 2 of the following criteria were fulfilled: 5-min Apgar score ≤ 7; moderate acidosis during the first hour of life: pH < 7.15 (umbilical artery, umbilical veins, capillary or arterial blood sample); and mild-to-moderate encephalopathy (Sarnat stage I—II) [16]. The present study used the World Health Organization (WHO) diagnostic criteria in combination with local guideline criteria: 1, Apgar score ≤ 7 at 1 min or 5 min, without the establishment of effective spontaneous respiration; 2, umbilical arterial blood pH < 7.15; and 3, exclusion of other causes of low Apgar score, such as premature infants. Low birth weight was defined as a birth weight < 2500 g. Postpartum hemorrhage was defined as an amount of bleeding ≥500 mL for vaginal delivery or ≥ 1000 mL for cesarean delivery within 24 h after delivery based on the International Federation of Gynecology and Obstetrics (FIGO) guideline [17]. Premature rupture of the membrane was defined as prelabor rupture of membranes that occurred 3 or more hours before preterm delivery. Preterm birth was defined as delivery of a newborn whose gestational age was < 37 weeks. HCA was defined as dense polymorphonuclear leukocyte infiltration of the amnion, chorion and decidua. Puerperal infection was defined as any infection that occurred during the puerperium, such as infection of the urogenital tract, breast, and respiratory system [18]. All pregnant women and children were followed up for 6 weeks. In-hospital or telephone follow-up every 7 days after delivery to ask about neonatal jaundice and weight gain, whether the mother had fever, mastitis, lochia, and recovery of the perineal wound. Complete blood counts of mother and newborn were performed at the six-week outpatient visit follow-up. Physical examination and bilirubin determination of newborns, and recovery of the uterus were also performed at six-week follow-up.

### Measurement of cytokines and chemokines

Within 24 h before delivery, peripheral blood was drawn for routine blood laboratory testing, and the WBCs, neutrophil ratio, and C-reactive protein (CRP) level were recorded. Cord blood was assessed for TNF-α, sICAM-1, and IL-8 using enzyme-linked immunosorbent assay kits. The microtiter plate was precoated with antibodies from the kit, and standards and samples were added to the wells of the plate and incubated for 2 h at 37 °C. After washing, the biotin-avidin-horseradish peroxidase system was used for color development. Tetramethylbenzidine was used as the substrate for enzyme-linked immunosorbent assay quantitative analysis to detect the absorbance at 450 nm. The levels of TNF-α, IL-8, and sICAM1 were determined via comparison with the standard curve.

### Sample size assessment

The sample size referred to a previously published study for the Chinese population, which reported an average positive rate of GBS of 13.89% during the 3-year follow-up. The probability of chorioamnionitis in GBS-positive patients was 16.93%. The probability of chorioamnionitis in GBS-negative patients was 8.86% [19]. According to the “pwr” package of R language, the required total sample size was 904 at 80% power and a significance level of 0.05. However, the incidence of HCA may be slightly lower than in this previous study because the study analyzed clinical and histological chorioamnionitis. The present study found that the positive rate of GBS was 15.9%, and the positive rates of HCA in GBS-positive and negative patients were 13.56 and 3.53%, respectively. The required total sample size was 283 under this condition. Therefore, we stopped the sample collection early.

### Statistical analysis

Quantitative data were first assessed to determine whether the data were consistent with a normal distribution. Normally distributed data are presented as the means ± standard deviation, and the two-sample t-test was used to analyze differences between groups. Non-normally distributed data are presented as the median (interquartile range), and the Kruskal-Wallis rank test was used to analyze differences between groups. The qualitative data were analyzed using the chi-squared test. Pearson’s correlation coefficient analysis was used to assess the correlation between variables. Univariate logistic regression was used to analyze the correlation between biomarker level and HCA. Multivariable analysis was also performed by adjusting for age, parity history and GBS infection. The receiver operating characteristic (ROC) curve was used to evaluate the accuracy of the diagnostic test and compare differences in the area under the ROC curve between the various biomarkers. All reported *P* values were two-sided, and *P* < 0.05 was considered significantly different. All analyses were performed using STATA software (version 14.0) and R project (version 3.6.3).

## Results

The present study enrolled 371 pregnant women in local hospitals from May 2019 to March 2020. Two women were excluded for early cesarean due to oligohydramnios, and two women were excluded for unexplained fever and diagnosed with listeria infection. Fifty-nine GBS-positive patients were in the GBS group, with a mean age of 29 ± 3.31 years, and 312 GBS-negative patients were in the non-GBS group, with a mean age of 29.57 ± 3.55 years. There was no significant difference in age or parity between the two groups. Pregnant women with GBS infection had a higher risk of pathological jaundice (*p* = 0.013), premature rupture of membranes (*p* = 0.043), and HCA (*p* = 0.001). There were higher levels of sICAM-1 (*p* < 0.001), IL-8 (*p* < 0.001), and TNF-α (*p* < 0.001) in the umbilical cord blood of GBS-infected mothers (Table 1).

**Table 1:** Characteristics of the included pregnant women grouped according to GBS infection

	GBS infection	Non-GBS infection	*p* value
No.	59	312	
Age	29 (3.31)	29.57 (3.55)	0.256
Median (Min-Max)	29 (21–36)	29 (22–40)	
Parity			
1	40	216	
2	18	85	
3	1	11	0.698
Pathologic jaundice	10	22	0.013
Non-reassuring fetal status	10	34	0.187
Birth asphyxia	6	20	0.300
Low birth weight infant	3	10	0.472
Postpartum hemorrhage	4	12	0.309
Premature rupture of membrane	8	19	0.043
Preterm birth	6	20	0.300
HCA	8	11	0.001
Puerperal infection	7	18	0.087
sICAM-1(ng/L)	354.5 (323.9–368.9)	291.3 (281.3–298.4)	< 0.001
IL-8(ng/L)	3573 (3287–3743)	1075.2 (1024.3–1276.4)	< 0.001
TNF-α (ng/L)	47.9 (45.8–50.1)	29.7 (28.7–30.8)	< 0.001
WBC(×10^9^)	8.72 (7.75–11.34)	8.675 (7.415–11.135)	0.764
Neutrophil ratio (%)	76 (73–83)	77 (73–81)	0.964
CRP (mg/L)	3.5 (2–10)	3.2 (2.5–5)	0.530

*Abbreviations*: *CRP* C-reation protein, *GBS* Group B Streptococcus, *HCA* Histological chorioamnionitis, *IL-8* Interleukin 8, *sICAM-1* soluble Intercellular adhesion molecule-1, *TNF-α* Tumor necrosis factor α, *WBC* White blood cells

Based on HCA status, the patients were classified into HCA and non-HCA groups. Nineteen patients had HCA with 8 GBS positive, and 352 patients did not have HCA. There was no significant difference between the two groups in age or parity. HCA in patients was associated with a higher risk of pathological jaundice (*p* = 0.005), fetal distress (*p* = 0.045) and low-birth-weight infants (*p* < 0.001). For parturients, there was also a higher risk of postpartum hemorrhage (*p* = 0.011), premature rupture of membranes (*p* = 0.001), and puerperal infection (*p* < 0.001). HCA patients had higher levels of sICAM-1 (*p* = 0.001), IL-8 (*p* < 0.001), and TNF-α (*p* < 0.001) in their cord blood and a higher WBC (*p* = 0.008), neutrophil ratio (< 0.001), and CRP (0.002) level in their peripheral blood (Table 2).

**Table 2:** Characteristics of the included pregnant women grouped according to HCA

	HCA	Non-HCA	*p* value
No.	19	352	
Age	28.63 (2.54)	29.52 (3.55)	0.282
Median (Min-Max)	29 (24–36)	29 (21–40)	
Parity			
1	17	239	
2	2	101	
3	–	12	0.135
GBS infection	8	51	0.001
Pathologic jaundice	5	27	0.005
Non-reassuring fetal status	5	39	0.045
Birth asphyxia	2	24	0.537
Low birth weight infant	4	9	< 0.001
Postpartum hemorrhage	3	13	0.011
Premature rupture of membrane	5	22	0.001
Preterm birth	2	24	0.537
Puerperal infection	10	15	< 0.001
SICAM-1(ng/L)	319.8 (296.3–376.4)	295.25 (283.3–307.15)	0.001
IL-8(ng/L)	1534.7 (1345.2–3528.1)	1120.6 (1025.1–1302.1)	< 0.001
TNF-α (ng/L)	37.1 (36.4–48.1)	30.1 (28.9–31.4)	< 0.001
WBC(×10^9^)	13.54 (7.37–15.38)	8.665 (7.475–10.895)	0.008
Neutrophil ratio (%)	84 (76–89)	77 (73–80.5)	< 0.001
CRP (mg/L)	12.3 (3.06–32.85)	3.2 (2.39–5.05)	0.002

*Abbreviations*: *CRP* C-reation protein, *GBS* Group B Streptococcus, *HCA* Histological chorioamnionitis, *IL-8* Interleukin 8, *sICAM-1* soluble Intercellular adhesion molecule-1, *TNF-α* Tumor necrosis factor α, *WBC* White blood cells

### Correlation analysis of multiple variables

**Fig. 1:**
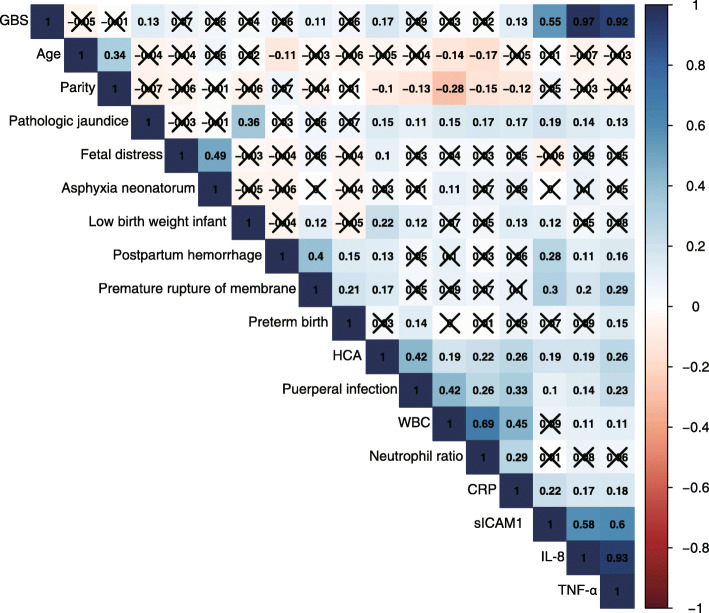
Correlation plot of the multiple variables included in this work. The results without significant differences are marked

All included variables were subjected to correlation analysis, and the results confirmed that GBS had a high correlation with IL-8 and TNF-α levels. There was also a significant correlation between IL-8 and TNF-α. There was a negative correlation between parity and peripheral blood WBC count. TNF-α and IL-8 also significantly correlated with pathological jaundice, postpartum hemorrhage, premature rupture of membranes, HCA, and puerperal infection (Fig. 1).

### Regression analysis

The regression analysis analyzed the relationship between biomarkers and HCA. Univariate analysis revealed that sICMA-1 (odds ratio (OR): 1.02; 95% CI: 1.01–1.03; *p* = 0.001), IL-8 (OR: 1.001; 95% CI: 1, 1.001; *p* = 0.001), and TNF-α (OR: 1.111; 95% CI: 1.06–1.165; *p* < 0.001) were significantly related to increased HCA risk. An increased WBC (OR: 1.211; 95% CI: 1.085–1.351; *p* = 0.001), neutrophil ratio (OR: 1.179; 95% CI: 1.088–1.278; *p* < 0.001), and CRP level (OR: 1.063; 95% CI: 1.028–1.098; *p* < 0.001) in peripheral blood were also significantly related to the occurrence of HCA (Table 3). After adjustments were made for age, parity, and GBS infection, a significant relationship between the biomarkers and HCA remained.

**Table 3:** Relationship between biomarker indicators and HCA by regression analysis

Indicators	Univariate analysis				Multivariate analysis^a^			
	Odd ratio	95% Confidence Interval		*p* value	Odd ratio	95% Confidence Interval		*p* value
sICAM1	1.020	1.010	1.03	0.001	1.018	1.004	1.033	0.015
IL-8	1.001	1.000	1.001	0.001	1.002	1.000	1.004	0.038
TNF-α	1.111	1.060	1.165	< 0.001	1.329	1.160	1.522	< 0.001
Neutrophil ratio	1.179	1.088	1.278	< 0.001	1.172	1.078	1.275	< 0.001
WBC	1.211	1.085	1.351	0.001	1.174	1.044	1.32	0.007
CRP	1.063	1.028	1.098	< 0.001	1.052	1.019	1.085	0.002

*Abbreviations*: *CRP* C-reation protein, *GBS* Group B Streptococcus, *HCA* Histological chorioamnionitis, *IL-8* Interleukin 8, *sICAM-1* soluble Intercellular adhesion molecule-1, *TNF-α* Tumor necrosis factor α, *WBC* White blood cells

^a^Adjust for adjusting for age, parity, and GBS infection

### ROC curve analysis

**Fig. 2:**
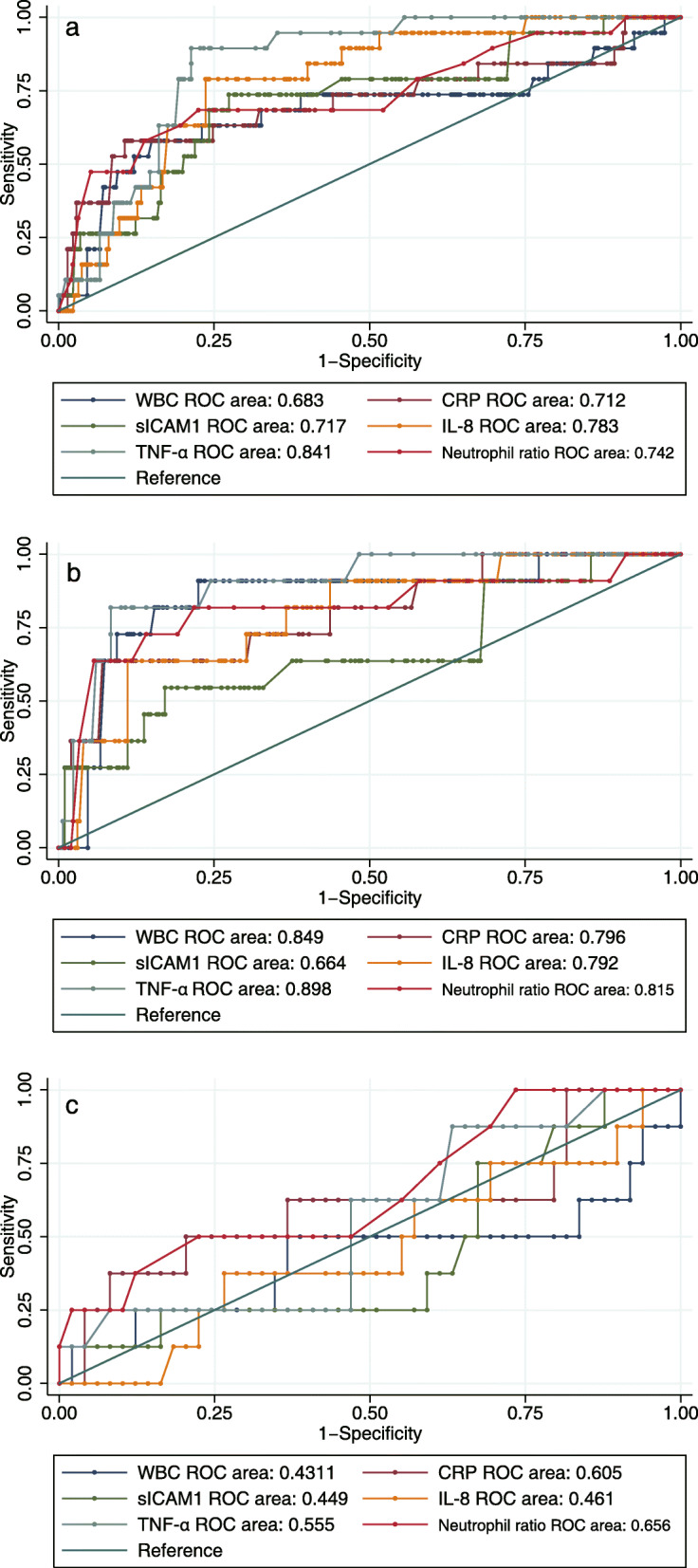
The ROC curves for HCA diagnosis with biomarkers. **a** Total pregnancy; **b** Pregnant without GBS infection; **c** Pregnant with GBS infection

ROC curves were used to analyze the diagnostic accuracy of the biomarkers for HCA. First, all of the populations were analyzed. Cord blood TNF-α had the largest ROC area (area under ROC curve: 0.841; 95% CI: 0.778–0.904) of the biomarkers. The cutoff value of TNF-α for the population was 32.7 ng/L with 89.47% sensitivity and 78.41% specificity (Fig. 2a). Subgroup analyses were performed according to whether GBS infection occurred. For the non-GBS infection population, TNF-α still had the largest area under the ROC curve (area: 0.898; 95% CI: 0.814–0.982) when the cutoff point was 32.7 ng/L with 81.82% sensitivity and 91.69% specificity. The neutrophil ratio also had an ideal area under the curve (area: 0.815; 95% CI: 0.645–0.985) with 63.64% sensitivity and 94.35% specificity at the 88% cutoff point, and peripheral blood WBC had an ideal area under the curve (area: 0.849; 95% CI: 0.72–0.978) with 81.82% sensitivity and 84.72% specificity at the cutoff point of 13.19 × 10^9^ and 63.64% sensitivity and 92.69% specificity at the 15.3× 10^9^ cutoff point (Fig. 2b). However, the biomarkers in the GBS-infected population had lower value in the diagnosis of HCA. Only CRP had a large area under the ROC curve (area: 0.605; 95% CI: 0.347–0.862) (Fig. 2c) (Table 4).

**Table 4:** The area under ROC curve for HCA diagnosis with biomarkers

Population	Biomarkers	No.	ROC area	95% Confidence Interval	
Total pregnant	TNF-α	366	0.841	0.778	0.904
	IL-8	366	0.783	0.693	0.873
	sICAM-1	366	0.717	0.590	0.844
	Neutrophil ratio	366	0.742	0.605	0.879
	WBC	366	0.683	0.524	0.842
	CRP	366	0.712	0.559	0.866
Non-GBS infection	TNF-α	309	0.898	0.814	0.982
	IL-8	309	0.792	0.659	0.925
	sICAM-1	309	0.664	0.469	0.858
	Neutrophil ratio	309	0.815	0.646	0.985
	WBC	309	0.849	0.721	0.977
	CRP	309	0.796	0.646	0.946
GBS infection	TNF-α	57	0.555	0.337	0.773
	IL-8	57	0.461	0.237	0.684
	sICAM-1	57	0.449	0.219	0.679
	Neutrophil ratio	57	0.656	0.435	0.876
	WBC	57	0.431	0.150	0.712
	CRP	57	0.605	0.347	0.862

*Abbreviations*: *CRP* C-reation protein, *GBS* Group B Streptococcus, *HCA* Histological chorioamnionitis, *IL-8* Interleukin 8, *sICAM-1* soluble Intercellular adhesion molecule-1, *TNF-α* Tumor necrosis factor α, *WBC* White blood cells

## Discussion

Chorioamnionitis is primarily caused by infections related to a variety of serious perinatal and neonatal adverse outcomes. However, typical clinical symptoms are rare, and many insidious cases of HCAs do not receive timely diagnosis and treatment, which adversely affects maternal and child health. However, ideal indicators for the diagnosis of HCA in prenatal examinations are lacking.

GBS is a common opportunistic pathogen that is routinely screened before delivery. Invasive GBS infection is also an important cause of neonatal abnormalities and mortality [20], and preterm birth, neonatal encephalopathy and stillbirth significantly correlate with GBS infection [21–23]. With the spread of antibiotic prophylaxis, the rate of invasive GBS infection decreased from 1.1 to 0.3% [24]. A recent systematic review showed an invasive GBS infection incidence of 0.49 per 1000 live births (95% CI, 0.43–0.56), in which EOD incidence was 0.41 (95% CI, 0.36–0.47) with 78% sepsis and 16% meningitis, and LOD was 0.26 (95% CI, 0.21–0.30) with 53% sepsis and 43% meningitis [25]. The mean case fatality ratio was 8.4 to 9.6% [25, 26]. A meta-analysis of the incidence of HCA in a population with normal delivery outcomes is lacking, but approximately 12.7% had HCA in one study based on Bangladeshi population [27], and the incidences of acute and chronic HCA were approximately 12.9 and 12.7%, respectively, in another study based on an American population [28]. HCA also significantly correlated with fetal adverse outcomes, including sepsis, bronchopulmonary dysplasia, intraventricular hemorrhage, necrotizing enterocolitis, and cerebral palsy [29–32]. The present study found that GBS infection significantly correlated with pathological jaundice, premature rupture of membranes, and HCA. Patients with HCA had a higher risk of pathological jaundice, fetal distress, and low-birth-weight infants. For parturients, there was also a higher risk of postpartum hemorrhage, premature rupture of membranes, and puerperal infection. For the biomarkers, TNF-α was accurate for the HCA diagnosis in non-GBS-infected pregnant women but not in the GBS-infected population. The neutrophil ratio and WBCs were also potential diagnostic biomarkers for HCA in the non-GBS infection population, which meets one of the diagnostic criteria for clinical chorioamnionitis.

The present study first confirmed that GBS infection increased TNF-α levels in cord blood. Mechanistically, GBS regulates the activation of the transcription factors NF-κB and AP-1 via the p38-MAPK pathway in human cord blood mononuclear cells to increase TNF-α expression and secretion [33]. TNF-α further activates the p38-MAPK pathway of neutrophils to promote the aggregation of neutrophils, which increases the risk of chorioamnionitis [34]. Although a clinical study suggested that TNF-α level was not significantly different in cord blood between pregnancies with and without clinical chorioamnionitis [35], our result was different, and this difference may be due to discrepant clinical and HCA diagnostic criteria. The present study suggests that TNF-α may help the diagnosis HCA, but whether an invasive measurement of TNF-α levels in cord blood is necessary to predict the risk of HCA requires further discussion.

The present study also found that an elevated TNF-α level, neutrophil ratio, and WBCs had more diagnostic accuracy for HCA in non-GBS-infected pregnant women. This result indicated that an elevated TNF-α level, neutrophil ratio, and WBCs caused by factors other than GBS infection may be important factors in causing HCA. Escherichia coli, GBS, and enterococcus species are the main types of infectious bacteria that affect birth outcomes [36]. Bacteria may vary between people in different regions. These bacteria may also increase the level of TNF-α in cord blood [37]. Another pathogen, human ureaplasma, also causes chorioamnionitis [38]. Intrauterine infection caused by ureaplasma was also related to TNF-a regulation in animal models [39]. GBS colonization lost its correlation with chorioamnionitis with routine GBS screening and prophylaxis in developed countries and even became negatively correlated [40]. Due to the preventive application of antibiotics in GBS-positive pregnant women, the probability of other opportunistic pathogen infections was reduced, which reduced the risk of HCA. Pregnant women with GBS infection also underwent routine antibiotic application in our study, which may explain why no diagnostic value was found for any examined biomarker for HCA. Therefore, whether to expand the antibiotic application standards to non-GBS-infected pregnant women with a high WBC, neutrophil ratio, or TNF-α level to reduce the risk of HCA needs further consideration.

In addition to the diagnostic value of TNF signaling, basic research showed that the inhibition of TNF signaling silenced the expression of 80% of infection-related genes that encode proinflammatory factors in response to lipopolysaccharides (LPS) and reduced the accumulation and activation of neutrophils at the feto-maternal interface [41]. Therefore, whether TNF signaling inhibition could prevent HCA in high-risk pregnant women to avoid serious adverse perinatal and neonatal outcomes requires further study.

In general, the diagnosis of clinical chorioamnionitis patients relies too heavily on the judgment of fever, which results in a 15% higher prevalence of clinical chorioamnionitis for HCA [42]. However, some patients with HCA lack the typical clinical manifestations [43]. This difference in diagnostic criteria for clinical chorioamnionitis and HCA results in heterogeneity between studies [44]. HCA is only confirmed via delayed pathological detection, but it was not achieved in every puerpera, which may lead to a missed diagnosis or loss of treatment opportunity. Therefore, biomarkers are needed to help diagnose HCA. A meta-analysis found that maternal CRP and WBC count showed low sensitivity and specificity for HCA diagnosis [45]. The present study found that the level of TNF-α detected from umbilical cord blood during delivery showed higher sensitivity and specificity that provided clues for HCA diagnosis. GBS colonization screening was generally performed, and antibiotics prophylaxis was used in positive populations. TNF-α had high diagnostic sensitivity and specificity in GBS-negative patients for HCA diagnosis. Therefore, TNF-α in cord blood may be measured at the same time as delivery. Increased maternal and infant monitoring and active antibiotic intervention should be performed as early as possible in patients with high levels of TNF-α (> 32.7 ng/L) to prevent HCA-related complications.

The present study had some limitations. First, the types of biomarkers were insufficient, and biomarkers, such as IL-6, were not studied. Second, we still need to follow local diagnostic criteria for clinical outcome description for clinical outcomes diagnostic criteria. The diagnostic criteria recommended in international guidelines are not fully followed. This difference will cause some error between the number of cases in the present work and the number of cases under international guidelines. Third, this work analyzed only pregnant women in the local population, and whether TNF-α has diagnostic accuracy for pregnant women in other regions and countries must be confirmed.

## Conclusion

In conclusion, GBS infection and HCA correlated with several perinatal and neonatal adverse outcomes, respectively. TNF-α in cord blood and the WBCs in peripheral blood had diagnostic value for HCA in non-GBS-infected pregnant women but not GBS-infected pregnant women.

## Supplementary Information

**Additional file 1.**

## Data Availability

The datasets used and/or analyzed during the current study are available from the corresponding author on reasonable request.

## Abbreviations

CRP: C-Reactive protein
GBS: Group B streptococcus
HCA: Histological chorioamnionitis
IL-8: Interleukin 8
OR: Odds ratio
ROC: Receiver operating characteristic
sICAM-1: soluble intercellular adhesion molecule-1
TNF-α: Tumor necrosis factor α
WBC: White blood cell count
