# Supplementary material for: Biomarkers for a histological chorioamnionitis diagnosis in pregnant women with or without group B streptococcus infection: a case-control study
Source: BMC Pregnancy Childbirth. 2021 Mar 25;21:250. doi: 10.1186/s12884-021-03731-7 (PMC7993527; doi:10.1186/s12884-021-03731-7)
Supplement: Supplementary file 1 — Additional file 1. [file 12884_2021_3731_MOESM1_ESM.docx]

| **Survey** | | | | | | | | |  |  |
| --- | --- | --- | --- | --- | --- | --- | --- | --- | --- | --- |
| Project Number: |  | |  | |  | | Project leader: |  |  |  |
| Name(or Participant ID) |  | | Sex | | Female | | Department | | |  |
| Age |  | | Occupation | |  | | Marital status | | |  |
| Race |  | | Address | |  | | Tel. | | |  |
| Past history |  | | Allergic history | |  | | Personal History | | |  |
| Data of admission |  | | Date of discharge | |  | | Number of Admission | | |  |
| Gestational age(Week) |  | | Parity | |  | | GBS infection detection | | |  |
| Vital sign | | | | | | | | | | |
| Temperature(℃) |  | | Pulse(Times/Min) | |  | | Respiratory rate(Times/Min) | | |  |
| Blood pressure(mmHg) |  | |  | |  | |  | | |  |
| Clinical diagnosis | | | | | | | | | | |
| Pathological jaundice |  | | Non-reassuring fetal status | |  | | Birth asphyxia | | |  |
| Low birth weight |  | | Preterm birth | |  | | Postpartum hemorrhage | | |  |
| Premature rupture of the membrane |  | | Histological chorioamnionitis | |  | | Puerperal infection | | |  |
| Others |  | | | | | | | | | |
| If the case is excluded,  please explain the reason |  | | | | | | | | | |
| Blood routine examination | | | | | | | | | | |
| WBC(×10^9^) |  | | Neutrophil ratio(%) | |  | | C-reactive protein(mg/L) | | |  |
| Cord blood examination | | | | | | | | | | |
| sICAM-1(ng/L) |  | | IL-8(ng/L) | |  | | TNF-α(ng/L) | | |  |
| Provider |  | Recorder | |  | | Record date | |  |  |  |
